# A high-transparency, micro-patternable chip for X-ray diffraction analysis of microcrystals under native growth conditions

**DOI:** 10.1107/S1399004715015011

**Published:** 2015-09-26

**Authors:** Thomas D. Murray, Artem Y. Lyubimov, Craig M. Ogata, Huy Vo, Monarin Uervirojnangkoorn, Axel T. Brunger, James M. Berger

**Affiliations:** a Biophysics Graduate Group, University of California, Berkeley, CA 94720, USA; b Department of Biophysics and Biophysical Chemistry, Johns Hopkins University School of Medicine, Baltimore, MD 21205, USA; c Departments of Molecular and Cellular Physiology, Neurology and Neurological Sciences, Structural Biology and Photon Science, and Howard Hughes Medical Institute, Stanford University, Stanford, CA 94305, USA; d GM/CA@APS, X-Ray Science Division, Advanced Photon Source, Argonne National Laboratory, Argonne, IL 60439, USA; e Department of Biomedical Engineering, Johns Hopkins University, Baltimore, MD 21205, USA

**Keywords:** microcrystals, silicon nitride, serial data collection, XFEL, microfocus beamline, X-ray crystallography, microfluidics

## Abstract

Microcrystals present a significant impediment to the determination of macromolecular structures by X-ray diffraction methods. Although microfocus synchrotron beamlines and X-ray free-electron lasers (XFELs) can enable the collection of interpretable diffraction data from microcrystals, there is a need for efficient methods of harvesting small volumes (<2 µl) of microcrystals grown under common laboratory formats and delivering them to an X-ray beam source under native growth conditions. One approach that shows promise in overcoming the challenges intrinsic to microcrystal analysis is to pair so-called ‘fixed-target’ sample-delivery devices with microbeam-based X-ray diffraction methods. However, to record weak diffraction patterns it is necessary to fabricate devices from X-ray-transparent materials that minimize background scattering. Presented here is the design of a new micro-diffraction device consisting of three layers fabricated from silicon nitride, photoresist and polyimide film. The chip features low X-ray scattering and X-ray absorption properties, and uses a customizable blend of hydrophobic and hydrophilic surface patterns to help localize microcrystals to defined regions. Microcrystals in their native growth conditions can be loaded into the chips with a standard pipette, allowing data collection at room temperature. Diffraction data collected from hen egg-white lysozyme microcrystals (10–15 µm) loaded into the chips yielded a complete, high-resolution (<1.6 Å) data set sufficient to determine a high-quality structure by molecular replacement. The features of the chip allow the rapid and user-friendly analysis of microcrystals grown under virtually any laboratory format at microfocus synchrotron beamlines and XFELs.

## Abstract 2

A highly X-ray-transparent, silicon nitride-based device has been designed and fabricated to harvest protein microcrystals for high-resolution X-ray diffraction data collection using microfocus beamlines and XFELs.

## 1. Introduction   

X-ray crystallography has long aided researchers in determining the high-resolution structures of biological macromolecules and their complexes. Although widespread in their use and utility, crystallographic efforts to obtain structural information are frequently hampered by an inability to obtain large, well diffracting crystals. Indeed, microcrystals (<15 µm in the longest dimension) are often found during initial searches for crystallization conditions, and in many instances projects can stall in proceeding beyond the microcrystal limit. The challenge of working with microcrystals has spurred the development of technical innovations aimed at overcoming the weak diffraction and radiation-sensitivity inherent to micrometre-sized samples. In recent years, advances such as microfocus synchrotron beamlines and X-ray free-electron lasers (XFELs) have made it possible to collect useful diffraction data from a variety of microcrystalline systems (Boutet *et al.*, 2012 ▸; Chapman *et al.*, 2006 ▸, 2011 ▸; Cowan & Nave, 2008 ▸; Cusack *et al.*, 1998 ▸; Finfrock *et al.*, 2010 ▸; Fischetti *et al.*, 2009 ▸, 2013 ▸; Hilgart *et al.*, 2011 ▸; Johansson *et al.*, 2012 ▸; Moukhametzianov *et al.*, 2008 ▸; Perrakis *et al.*, 1999 ▸; Sanishvili *et al.*, 2008 ▸, 2011 ▸; Sawaya *et al.*, 2014 ▸; Smith *et al.*, 2012 ▸).

Although beamline technologies can presently work with crystals in the submicrometre size regime (Chapman *et al.*, 2011 ▸), sample harvesting and targeting remain two persistent difficulties associated with microcrystal diffraction studies. For example, with XFELs, liquid-jet injection systems can be used to continuously flow a thin stream of microcrystals into the path of a rapidly pulsing X-ray beam (DePonte *et al.*, 2008 ▸; Sierra *et al.*, 2012 ▸; Weierstall *et al.*, 2012 ▸, 2014 ▸). Liquid jets have successfully been used in several structural analyses (Barends *et al.*, 2014 ▸; Botha *et al.*, 2015 ▸; Boutet *et al.*, 2012 ▸; Chapman *et al.*, 2011 ▸; Johansson *et al.*, 2013 ▸; Kern *et al.*, 2012 ▸, 2013 ▸, 2014 ▸; Kupitz *et al.*, 2014 ▸; Liu, Wacker *et al.*, 2013 ▸; Redecke *et al.*, 2013 ▸); however, these devices also have an intrinsically low ‘hit’ rate (*i.e.* a low number of ‘beam-on-target’ events compared with the total number of exposures collected) and they tend to consume large sample volumes (hundreds of microlitres to tens of millilitres) during the course of an experiment (Boutet *et al.*, 2012 ▸; Chapman *et al.*, 2011 ▸; Sierra *et al.*, 2012 ▸; Weierstall *et al.*, 2014 ▸). Capillary-based liquid-stream systems, which have successfully been used with microfocus synchrotron beamlines (Stellato *et al.*, 2014 ▸), have similarly large sample requirements.

‘Fixed-target’ devices for delivering microcrystals constitute a valuable alternative to liquid-jet systems (Cohen *et al.*, 2014 ▸; Hirata *et al.*, 2014 ▸). Loops, micromeshes or polycarbonate plastic grids offer low volume consumption and relatively high target hit rates (Cohen *et al.*, 2014 ▸; Hirata *et al.*, 2014 ▸), a useful feature in instances when crystalline material is limiting. Despite these beneficial properties, fixed-target devices are not without their own drawbacks. For example, crystals deposited onto plastic meshes require cryopreservation to avoid dehydration; the search for suitable cryoprotection conditions is often difficult, and in some cases the change in solutes or the cooling process itself can adversely reduce the crystalline order or affect the protein structure (Fraser *et al.*, 2009 ▸, 2011 ▸; Keedy *et al.*, 2014 ▸; Tilton *et al.*, 1992 ▸). Some fixed-target delivery systems, such as MiTeGen MicroRT capillary mounts, PDMS-based microfluidic devices (Heymann *et al.*, 2014 ▸; Khvostichenko *et al.*, 2014 ▸; Lyubimov *et al.*, 2015 ▸), silicon nitride grids (Frank *et al.*, 2014 ▸; Hunter *et al.*, 2014 ▸; Kimura *et al.*, 2014 ▸), the HC1 humidity-control device (Sanchez-Weatherby *et al.*, 2009 ▸) and polyimide (Kapton) film-based grids (Cipriani *et al.*, 2012 ▸; Zarrine-Afsar *et al.*, 2012 ▸) can circumvent problems associated with cryoprotection by permitting sample analysis at room temperature. Furthermore, microfluidic devices can allow the growth or capture of microcrystals from dilute solutions within addressable arrays (Hansen *et al.*, 2002 ▸, 2006 ▸; Heymann *et al.*, 2014 ▸; Khvostichenko *et al.*, 2014 ▸; Lyubimov *et al.*, 2015 ▸; Zheng *et al.*, 2004 ▸).

One disadvantage that some fixed-target approaches suffer compared with liquid-jet devices is an increase in background scatter that arises from the device material. For example, PDMS gives rise to a sharp scattering ring at ∼7.5 Å resolution (Dhouib *et al.*, 2009 ▸; Greaves & Manz, 2005 ▸; Guha *et al.*, 2012 ▸) and device fabrication typically requires relatively thick (>50 µm) material layers, properties that can interfere with weak diffraction patterns (Hansen *et al.*, 2006 ▸; Lyubimov *et al.*, 2015 ▸). Silicon nitride supports offer relatively low background scatter and high X-ray transmission compared with PDMS (Frank *et al.*, 2014 ▸; Hunter *et al.*, 2014 ▸), but microcrystal-harvesting devices of this type reported thus far have relied on loading schemes that remove samples from their native growth conditions (Feld & Frank, 2014 ▸; Frank *et al.*, 2014 ▸; Hunter *et al.*, 2014 ▸), a step that can adversely impact diffraction quality. The HC1 humidity-control device allows room-temperature data collection through user-controllable partial dehydration of crystalline samples on thin mounts; however, the system requires that optimized crystals be cryocooled for full data-set collection (Sanchez-Weatherby *et al.*, 2009 ▸). For their part, polyimide (Kapton) film based-devices have been described that exhibit low scattering and that can maintain native crystal-growth conditions during loading (Cipriani *et al.*, 2012 ▸; Zarrine-Afsar *et al.*, 2012 ▸); however, these systems have either required that crystals be grown in the device (Cipriani *et al.*, 2012 ▸) or that large glass beads (Zarrine-Afsar *et al.*, 2012 ▸) whose dimensions are much larger (up to 100 µm) than those of the microcrystals be used to create a textured surface for randomizing the crystal orientation (Zarrine-Afsar *et al.*, 2012 ▸).

To begin to overcome some of the limitations present in current fixed-target devices, a new silicon nitride-based chip that permits the collection of X-ray diffraction data from microcrystals under native growth conditions has been developed. Using a combination of high X-ray transmission materials, including silicon nitride, Kapton film and patternable photoresist, the chip is capable of accepting a small volume (<2 µl) of microcrystalline slurry from a standard pipette, confining it within a defined imaging area and favorably biasing the positions of single microcrystals with micro-patterned features. These chips were used to collect X-ray diffraction data under an XFEL-like, zero-oscillation, ‘one-crystal/one-shot’ strategy from ∼10–15 µm-sized hen egg-white lysozyme (HEWL) crystals on microfocus beamlines using manual targeting. This approach allowed the collection of a complete, high-quality (1.55 Å resolution) data set from <325 microcrystals, Additionally, test exposures of HEWL microcrystals using an X-ray free-electron laser (XFEL) produced high-quality diffraction data, demonstrating the utility of the chips for use with either microfocus synchrotron or XFEL X-ray radiation.

## 2. Materials and methods   

### 2.1. Chip fabrication   

100 mm diameter, 525 (±25) µm thick ‘prime’-grade silicon wafers coated with 1500 Å (±5%) of low-pressure, chemical vapor-deposited silicon nitride (WRS Materials) were used to fabricate silicon nitride windows for all device designs. Wafers were dehydration-baked at 200°C for 60 min and, following cooling, were patterned with 2 µm thick S1813 photoresist (Dow) into the desired arrays using a laser-etched chrome mask (Front Range PhotoMask) and standard photolitho­graphy techniques (Jaeger, 2002 ▸). S1813 photoresist was spun onto the back side of the wafer to a thickness of 2 µm to protect the silicon nitride layer on the back side during subsequent deep reactive ion etching (DRIE) treatment. Following a 115°C bake to harden the photoresist on the back side of the wafer, the wafer underwent DRIE using carbon tetrafluoride (CF_4_) plasma to selectively remove silicon nitride that was exposed from the previous transfer of the desired window pattern. The photoresist was then stripped off in an acetone bath and the wafer was then dunked into a 90°C 30%(*w*/*v*) potassium hydroxide bath for wet etching of the silicon wafer. The wet etch proceeded at a rate of roughly 1 µm min^−1^ and, following its completion, the wafer was then rinsed thoroughly with deionized water. To prepare the wafer for photoresist patterning of the middle layer, the wafer was dehydration-baked at 200°C for 60 min and was then oxygen plasma-treated (300 W for 5 min) using a PEII-A plasma etch chamber (Technics). SPR 220-7.0 photoresist (Dow) was subsequently spun onto the continuous silicon nitride surface side of the wafer to a height of 15 µm for channel chip designs, or SPR 220-3.0 (Dow) was spun to a height of 5 µm onto the wafer for photoresist-stripe chip designs. The photoresist features were patterned using standard photolithography techniques and a laser-etched chrome mask (Front Range PhotoMask). To render the photoresist stripes hydrophilic, the photoresist was oxygen plasma-treated using an Atomflo 400 series plasma generator (Surfx). Individual devices were excised from the wafer using a diamond-tipped pen to apply pressure along dice lines that were patterned onto the wafer along with the initial window array and etched into the wafer during wet etching.

### 2.2. Crystal growth and chip loading for optical imaging of microcrystals within silicon nitride chips   

Hen egg-white lysozyme (HEWL) microcrystals were grown as described by Falkner *et al.* (2005 ▸), except that a commercially available preparation of HEWL (OmniPure, 5950-OP) was used and the crystal cross-linking step was omitted. Lyophilized lysozyme was resuspended at 20 mg ml^−1^ in sodium acetate pH 3.5 and split into 50 µl aliquots that were then stored at −20°C. For crystallization, the lysozyme solution was thawed and then spun for 10 min at 13 000 rev min^−1^ in a bench-top centrifuge (Eppendorf 5424) to remove any precipitate or fine-particulate matter. The clarified solution was then transferred to a fresh microcentrifuge tube and crystallization buffer was added at a 1:3 (protein:buffer) ratio. To generate crystals of 10–15 µm in size, a 20% sodium chloride, 8%(*w*/*v*) poly(ethylene glycol) 8000, 0.5 *M* sodium acetate pH 3.5 solution was used. Crystals formed within 15 min when the solution was placed on ice or in a 4°C cold room.

For optical imaging of chips loaded with HEWL microcrystals, the microcrystalline slurry was first agitated by pipetting the mixture up and down. Next, 0.5 µl of this preparation was spotted on top of a photoresist-patterned silicon nitride window. For covering chips, a piece of 8 µm thick Kapton film (SPEX SamplePrep, catalog No. 3511) of sufficient dimensions to cover all silicon nitride observation windows was cut using a razor blade and placed on top of the solution. Images of crystals on the chip were taken using an Olympus IX71 microscope fitted with a Retiga 2000R camera (Q Imaging).

For X-ray data collection on Stanford Synchrotron Radiation Laboratory (SSRL) beamline BL12-2 and the X-ray Pump Probe (XPP) endstation of the Linac Coherent Light Source (LCLS), chips were loaded and covered as described for optical imaging. At the Advanced Photon Source, 0.5 µl freshly prepared HEWL microcrystal slurry was pipetted onto four consecutive windows, as only four windows were accessible for data collection on each silicon nitride chip owing to the geometry of the mounting devices and beamline end­station.

### 2.3. Diffraction data collection and crystal structure determination   

Background-scattering X-ray diffraction experiments were performed on SSRL beamline BL12-2 with a 10 µm beam, 3 s exposures and 0.02° oscillations. X-ray diffraction experiments on 10–15 µm HEWL microcrystals used a 10 µm beam, 5 s exposures and 0.02° oscillations. Images were collected on a Dectris PILATUS 6M detector. 5 × 30 mm channel-based chips (Supplementary Fig. S1) were used for all measurements. A custom-made chip mount was used for all experiments. Crystals positioned either entirely on a silicon nitride surface or entirely on a photoresist-coated silicon nitride surface produced diffraction without any noticeable differences (Supplementary Fig. S2). Both diffraction images were subsequently indexed with the expected space group and unit-cell parameters for tetragonal HEWL microcrystals (*P*4_3_2_1_2, *a* = *b* = 79.1, *c* = 38.1 Å, α = β = γ = 90.0°).

X-ray diffraction experiments were performed using 10–15 µm HEWL microcrystals at the XPP endstation of the LCLS with a 3 µm unattenuated beam and 40 fs pulses. Test exposures of 5 × 30 mm channel-based chips (Supplementary Fig. S1) were taken using either 40 fs pulses of a 3 µm un­attenuated beam (Supplementary Figs. S3*a*, 4*a* and 4*b*) or a 30 µm unattenuated beam (Supplementary Figs. S4*c* and S4*d*) at 9 keV. Still (zero-oscillation) images were collected on a Rayonix MX325 detector. 5 × 30 mm channel-based chips (Supplementary Fig. S1) and a custom-made chip mount (a standard magnetic base with a machined chip-holding slot) were used for all measurements. The detector was positioned such that the corner corresponded to a resolution of ∼2.0 Å. Using *JBluIce*–*EPICS* (Stepanov *et al.*, 2011 ▸), crystals were moved into the beam by first focusing on a single 1 mm^2^ silicon nitride window at low magnification. Subsequently, the magnification was increased to such a level that individual microcrystals could clearly be seen, and a single microcrystal was then manually targeted for exposure.

Collection of a full data set took place on the The General Medical Sciences and Cancer Institutes Structural Biology Facility (GM/CA) beamline 23-ID-D at the Advanced Photon Source (APS) using a zero-oscillation, serial data-collection strategy with a 10 µm diameter beam and a PILATUS3 6M detector. Chips were mounted using a custom-made chip holder developed at GM/CA. A total of 324 (0.25 s exposure) images were collected during <8 h of beamtime. Eight chips were used for data collection, with chips generally remaining mounted and under observation for 30–60 min in duration; one chip was examined for 107 min. HEWL microcrystals were located by eye by manually rastering from the upper left-hand to lower right-hand corner of a silicon nitride window. After exposing crystals within a single window, the chip was translated to the adjacent window, and this window was also rastered manually. Hydrophilic photoresist stripe-based chips (10 × 30 mm device dimensions, stripe-patterned) were used in these experiments.

To establish the minimum X-ray dose necessary for maximum-resolution data-set collection, a single HEWL microcrystal was first subjected to a series of ten 1.0 s exposures of radiation to determine the time at which the diffraction resolution limit began to decrease. After a single microcrystal survived only one 1.0 s exposure, a lower dose series of ten 0.3 s exposures were performed on another single microcrystal. The resolution decreased during the second 0.3 s exposure. After ten 0.1 s exposures revealed that the resolution limit did not extend to the edge of the detector, a 0.25 s exposure was chosen for data-set collection.

A total of 324/324 (0.25 s exposure) diffraction images were indexed and integrated using the *cctbx.xfel* software suite (Hattne *et al.*, 2014 ▸). *PRIME* was then used to successfully merge, scale and post-refine 324/324 of the integrated images (Uervirojnangkoorn *et al.*, 2015 ▸). The lysozyme structure was determined by molecular replacement using *Phaser* (McCoy *et al.*, 2007 ▸) and refined using *phenix.refine* (Adams *et al.*, 2010 ▸), while manual rebuilding and adjustment was carried out in *Coot* (Emsley *et al.*, 2010 ▸). *R*_free_-flagged reflections were withheld from all output maps, beginning with the molecular-replacement maps.

## 3. Results   

### 3.1. Construction of a three-layer silicon nitride-based chip for harvesting protein microcrystals   

**Figure 1:**
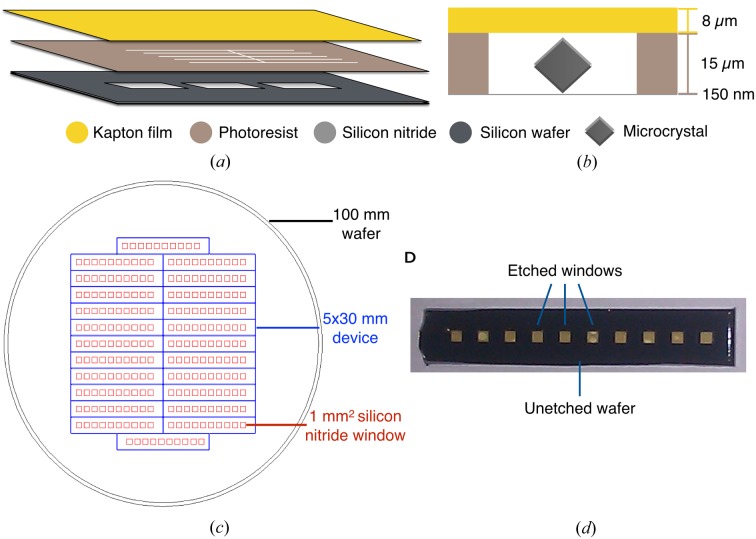
Three-layer device layout. (*a*) Schematic representation of chip layers. (*b*) Cut-away view of the imaging chamber of the assembled chip with a 150 nm thick silicon nitride window, 15 µm thick photoresist and 8 µm thick Kapton film layer. (*c*) 100 mm diameter wafer patterned with 24 5 × 30 mm devices. (*d*) Photograph of a single 5 × 30 mm device (silicon nitride layer only).

To construct a platform for microcrystal X-ray diffraction experiments, a three-layer chip was designed in which the top and bottom segments are composed of highly X-ray-transparent materials and the middle layer is configured as a separating support layer that also helps to trap microcrystalline samples (Figs. 1 ▸ *a* and 1 ▸ *b*). Silicon nitride was selected as the foundation material for the chip owing to its low cost and its established utility in diffraction experiments (Feld & Frank, 2014 ▸; Frank *et al.*, 2014 ▸; Hunter *et al.*, 2014 ▸; Kimura *et al.*, 2014 ▸). After consulting with staff scientists at the SSRL, the APS and the LCLS about desirable dimensions, a series of rectangular (5 × 30 mm) ‘chips’, which feature an array of ten 1 mm^2^ × 150 nm thick silicon nitride observation windows, were fabricated. For high fabrication efficiency, patterning masks that allow up to 24 such devices to be generated from a single 100 mm diameter silicon nitride-coated silicon wafer were created (Figs. 1 ▸ *c* and 1 ▸ *d*). Photoresist was chosen as a middle, supporting layer owing to its patternability and favorable X-ray transmission properties (Dhouib *et al.*, 2009 ▸; Wells, 1992 ▸). Commercially available 8 µm thick polyimide (Kapton) film was selected as the top layer for its low air permeability, frequent use in X-ray diffraction experiments (Cipriani *et al.*, 2012 ▸; Heymann *et al.*, 2014 ▸; Zarrine-Afsar *et al.*, 2012 ▸) and durability.

Photoresist patterning is highly tunable, allowing structures of virtually any shape to be constructed on a surface through standard photolithography (Jaeger, 2002 ▸). To help to confine and position protein microcrystals, the photoresist (middle) layer was initially patterned into a series of long parallel channels. The channels were designed to be 20 µm wide and 15 µm tall (corresponding to a set of dimensions somewhat larger than a typical microcrystal), separated by ∼50–100 µm wide photoresist walls and of a length that either spanned the entirety of the 1 mm^2^ silicon nitride window (Supplementary Fig. S1*a*) or that was divided into two 0.5 mm segments (Supplementary Fig. S1*b*). The relative channel-to-channel spacing was also varied in early prototypes of our chips (Supplementary Figs. S1*c* and S1*d*), although neither variation in channel length nor in channel spacing was subsequently found to affect chip performance (not shown).

**Figure 2:**
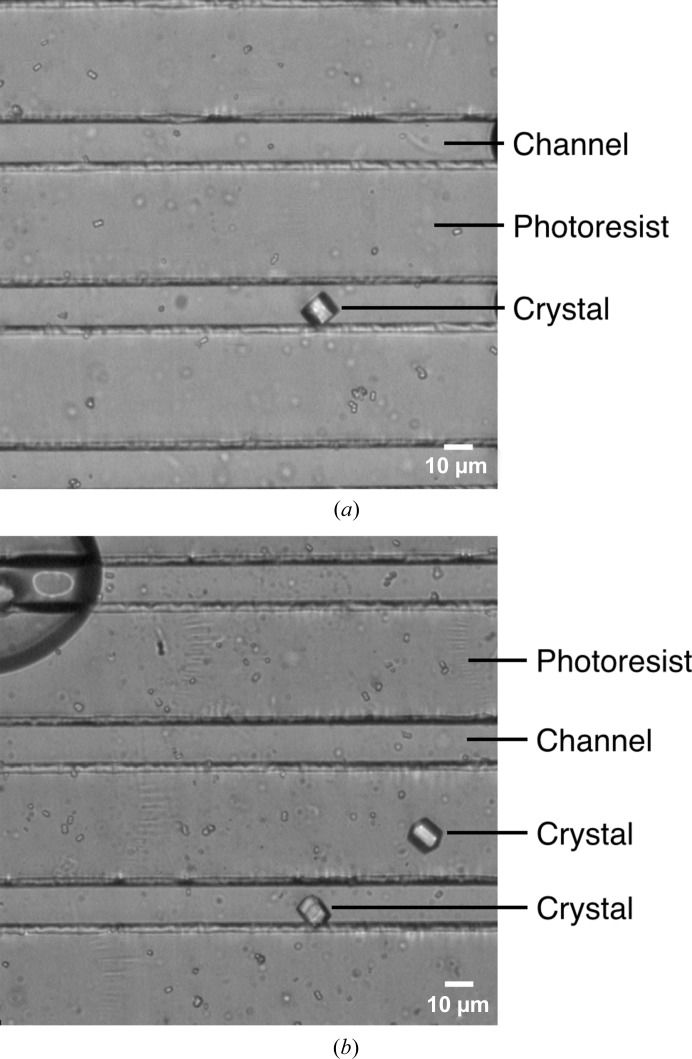
Optical imaging of loaded channel-based chips. (*a*, *b*) Light micrographs of sealed three-layer chips designed with 20 µm wide channels and loaded with 10–15 µm HEWL microcrystals.

To test the channel-based chip designs, 0.5 µl of a hen egg-white lysozyme (HEWL) microcrystalline slurry (∼10–15 µm average crystal size) was pipetted atop a photoresist-patterned silicon nitride window and the sample was covered with a single strip of Kapton film. Following the placement of the Kapton film layer, the crystals did indeed settle into the patterned channels (Fig. 2 ▸ *a*). However, crystals frequently resided on the topmost surface of the photoresist, sandwiched between this layer and the Kapton film (Fig. 2 ▸ *b*). Although test exposures did not reveal any appreciable increase in background scattering from chip areas that contained photoresist *versus* those that did not (Supplementary Fig. S2), the chips were re­designed in such a way that the crystals would favor either an association with the silicon nitride directly or with the edges of the photoresist features, so that the Kapton film did not press them into the photoresist.

### 3.2. Hydrophilic surface patterning improves microcrystal localization   

Based on the initial finding that crystals tended to associate with the upper, flat surface of the photoresist as readily as with the silicon nitride flooring of the patterned channels, the innately hydrophobic nature of both layers appeared to be preventing any preferential localization to one material or the other. This idea suggested that a relatively hydrophilic surface to which protein microcrystals could become stably anchored might be beneficial. To overcome the hydrophobicity of the entirety of the chip, an oxygen plasma treatment was used to render the photoresist layer hydrophilic (Northen & Turner, 2005 ▸; Walther *et al.*, 2007 ▸). A more textured surface to the chip was introduced by patterning denser numbers of photoresist structures onto the silicon nitride layer; such an approach has been shown to aid surface wetting (Marinaro *et al.*, 2014 ▸; Zarrine-Afsar *et al.*, 2012 ▸) and to help to randomize the orientation of samples deposited on a surface (Zarrine-Afsar *et al.*, 2012 ▸).

**Figure 3:**
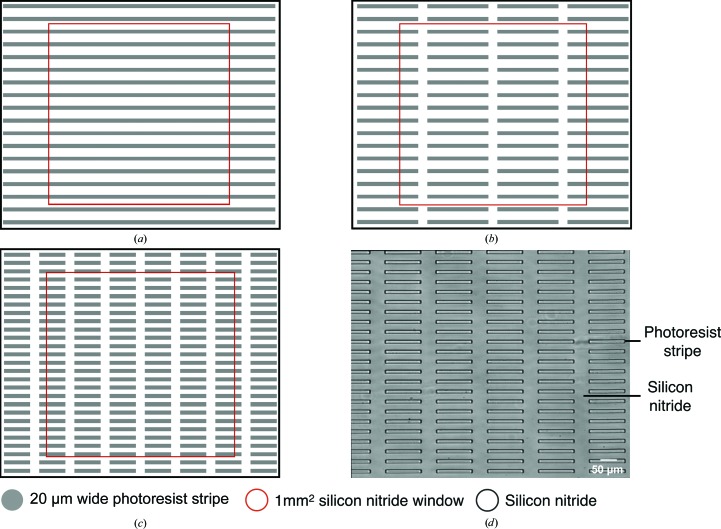
Photoresist stripe-based chip designs. (*a*, *b*, *c*) Schematic representation of the design masks for patterning a 1 mm^2^ silicon nitride window with 20 µm wide photoresist structures of varying lengths. (*d*) Optical micrograph of an area within a single 1 mm^2^ silicon nitride window patterned with 20 µm wide by 137.5 µm long photoresist structures.

**Figure 4:**
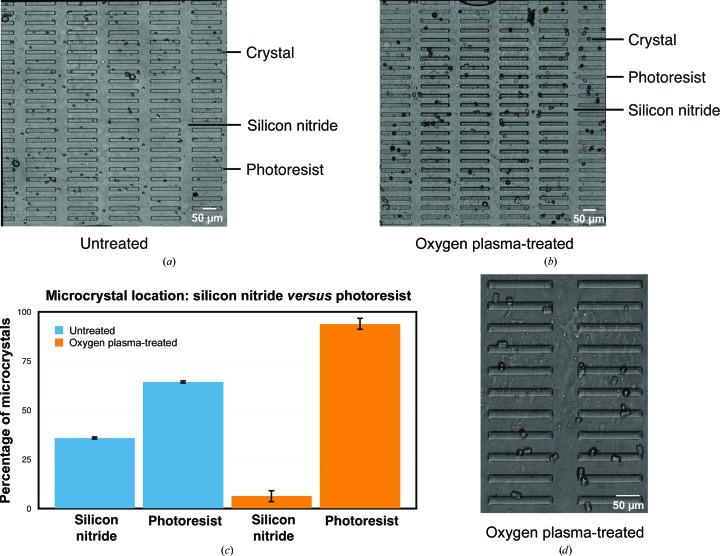
Optical imaging of HEWL microcrystals sealed in untreated and oxygen plasma-treated three-layer chips. (*a*) Light micrograph of a 1 mm^2^ silicon nitride window patterned with 20 µm wide, untreated photoresist stripes loaded with HEWL microcrystals. (*b*) Light micrograph of a 1 mm^2^ silicon nitride window patterned with 20 µm wide, oxygen plasma-treated photoresist stripes loaded with HEWL microcrystals. (*c*) Quantification of HEWL microcrystal number optical imaging within the bounds of a 1 mm^2^ silicon nitride window. (*d*) Close-up view of crystals within an oxygen plasma-treated chip.

Firstly, an array of rectangular photoresist stripes of varying dimensions and spacings was designed (Fig. 3 ▸). Stripes were 20 µm in width, and ranged from the length of the window to ∼150 µm in length. Following photoresist patterning and oxygen plasma treatment, light microscopy was used to assess whether microcrystals would preferentially associate with the edges of the treated photoresist stripes compared with untreated stripes. 0.5 µl of a freshly prepared HEWL microcrystal slurry was loaded onto a photoresist stripe-patterned silicon nitride window, covered with Kapton film and the number of observable crystals within the bounds of the 1 mm^2^ silicon nitride observation window was then counted (Figs. 4 ▸ *a* and 4 ▸ *b*). HEWL microcrystals showed a clear preference for localizing to the long edges of hydrophilic photoresist stripes (Fig. 4 ▸ *c*), indicating that that the features were indeed useful for biasing microcrystal placement. The more textured photoresist structures also appeared to help to randomize the orientation of microcrystals within the chip (Fig. 4 ▸ *d*).

### 3.3. X-ray diffraction studies of microcrystals deposited in silicon nitride chips   

**Figure 5:**
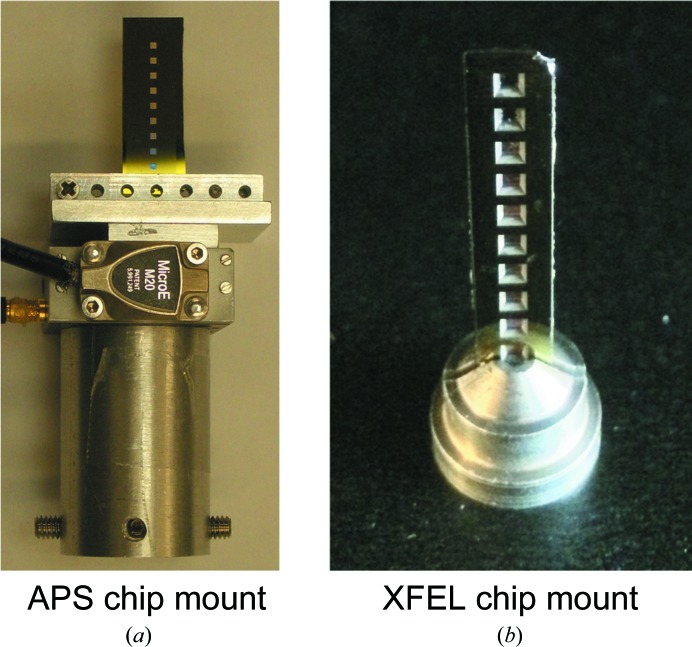
Chip holders used for X-ray diffraction experiments. (*a*) Image of a custom-made chip mount used on GMCA beamline 23-ID-D at the APS that is capable of holding chips of a variety of widths (a 10 × 30 mm device is pictured). (*b*) Image of a custom-made chip mount used at the XPP endstation of the LCLS.

To test the performance of the chips, they were assessed on the BL12-2 microfocus beamline at the SSRL using a mount derived from a standard crystal loop magnetic base. X-ray diffraction experiments were also conducted at the APS using a custom-built chip mount intended to hold devices of various sizes (Fig. 5 ▸ *a*) and at the XPP endstation of the LCLS using a modified magnetic base compatible with a standard gonio­meter (Fig. 5 ▸ *b*). The rigid nature of the chips allowed efficient mounting using a variety of custom-made holders in the confined space of a goniometer setup. The low total chamber thickness (<25 µm; Fig. 1 ▸ *b*) and optical transparency of the chips also allowed easy identification and targeting of crystals positioned within a silicon nitride observation window during data collection, even when the device was tilted by up to 45°.

**Figure 6:**
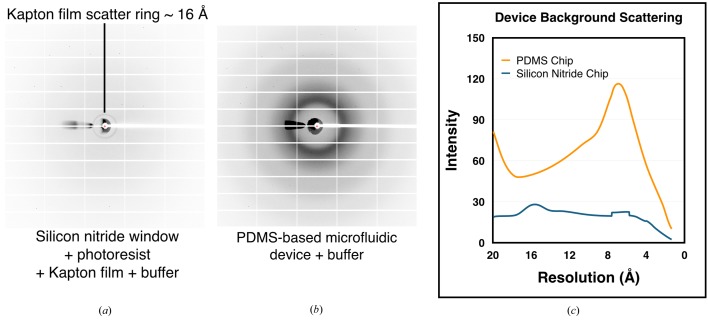
Comparison of background scattering between silicon nitride-based and PDMS-based devices. (*a*) Diffraction image from a three-layer (silicon nitride window, 15 µm thick photoresist and Kapton film) device containing crystallization buffer. The dark scattering ring at 16 Å resolution derives from the Kapton film. (*b*) Diffraction image from a ∼100 µm thick two-layer microfluidic PDMS crystal capture device loaded with crystallization buffer. (*c*) Plot of scattering intensity *versus* resolution for the images shown in (*a*) and (*b*). The cone-shaped dark area to the left of the beamstop corresponds to the part of the diffraction image where X-rays were not blocked by the X-ray scatter guard. Data for (*c*) were smoothed to remove sharp features caused by these unblocked X-rays.

As anticipated, the three-layer chips exhibited very low background signal (Fig. 6 ▸ *a*). The levels of background scatter proved to be much lower than that of a PDMS-based microfluidic crystal-capture device (Figs. 6 ▸ *b* and 6 ▸ *c*), with the only prominent background feature being a scattering ring generated by the Kapton film at ∼16 Å (Figs. 6 ▸ *a* and 6 ▸ *c*). The chips were next tested using ∼10–15 µm-sized HEWL microcrystals on BL12-2 at SSRL (Supplementary Fig. S2), the LCLS XPP endstation (Supplementary Fig. S3*a*) and GM/CA beamline 23-ID-D at the APS (Supplementary Fig. S3*b*). In each instance, the resultant diffraction images indexed with the space group (*P*4_3_2_1_2) and unit-cell parameters (*a* = *b* = 79.1, *c* = 38.1 Å, α = β = γ = 90°) commonly observed for tetragonal HEWL crystals. Moreover, high-resolution data were clearly evident in the diffraction pattern, with diffraction maxima frequently seen extending to ∼1.5 Å resolution (Supplementary Fig. S3*b*), compared with the ∼1.8 Å resolution spots that were occasionally observed from thin-layer PDMS chips (Lyubimov *et al.*, 2015 ▸).

### 3.4. Structural validation of chip performance   

Having established that the three-layer silicon nitride-based chips allow the collection of high-resolution diffraction data, a complete diffraction data set was collected using the devices at room temperature. Diffraction images were collected on GM/CA beamline 23-ID-D at the APS using ∼10–15 µm-sized HEWL microcrystals and a 10 µm diameter beam size. Total data-collection times often reached 1 h per chip, with the longest duration being 107 min from the first to last exposure; this amount of time proved to be more than sufficient to allow all segments of a chip to be used before the samples dehydrated. One zero-oscillation (‘still’) diffraction image per crystal was taken to approximate an XFEL-like serial data-collection experiment. Prior to data collection, the minimal X-ray exposure time (0.25 s) that would still yield maximum diffraction resolution for these crystals was determined (§2.3, Materials and Methods[Sec sec2.3]). This exposure time corresponds to approximately 0.05–0.1 times the number of 8 keV photons delivered in a single FEL pulse. Chip tilts (±44° in 2° increments) were used during collection to ensure that all regions of reciprocal space were appropriately sampled; in this regard, the hydrophilic and textured features of the photoresist proved valuable in randomizing microcrystal orientation, thereby allowing a complete serial X-ray diffraction data set to be collected from a relatively small (<325) number of microcrystals (Supplementary Fig. S5). The final data set was assembled from 324/324 of the still images acquired from eight photoresist stripe-patterned chips (Fig. 3 ▸). Interestingly, individual images often indexed and integrated to ∼1.3 Å resolution, demonstrating the high signal-to-noise properties of the chips (Supplementary Fig. S6).

Following data collection, *cctbx.xfel* (Hattne *et al.*, 2014 ▸) was used for indexing and integration of diffraction images and *PRIME* (Uervirojnangkoorn *et al.*, 2015 ▸) was used for scaling, merging and post-refinement. A total of 324 indexed/integrated diffraction images resulted from this process. The merged data set was 91.6% complete in the highest resolution bin (1.55 Å) and 96.9% complete overall (higher resolution data to 1.3 Å were not included in the refinement owing to low completeness). Chip tilting and random distribution of crystal orientations on the chip surface appeared to greatly aid the overall completeness, as a PDMS-based device used with a similar data-collection strategy still exhibited a missing wedge of diffraction data (Supplementary Fig. S5; Lyubimov *et al.*, 2015 ▸).

After merging, *Phaser* (McCoy *et al.*, 2007 ▸) was used for molecular replacement with a polyalanine search model derived from a HEWL structure (PDB entry 1vdp) crystallized in a different space group (*P*2_1_; S. Aibara, A. Suzuki, A. Kidera, K. Shibata, T. Yamane, L. J. DeLucas & M. Hirose, unpublished work). Only a single solution with an excellent log-likelihood gain (1279.3) and translation-function *Z*-score (39.0) resulted from this approach. MR-derived 2*mF*_o_ − *DF*_c_ electron-density maps clearly revealed most side chains and all disulfide bonds (Supplementary Fig. S7*a*). The structure was refined with *PHENIX* (Adams *et al.*, 2010 ▸), with manual intervention between refinement cycles carried out using *Coot* (Emsley *et al.*, 2010 ▸). The final model has good geometry as reported by *MolProbity* (Chen *et al.*, 2010 ▸) and closely matches reported tetragonal HEWL structures (Table 1 ▸, Supplementary Figs. S7*c* and S7*d*).

## 4. Discussion   

During the crystallization of biological macromolecules, it is not uncommon for researchers to obtain microcrystals. Indeed, for many projects, crystals on the order of 5–10 µm or less are all that can be obtained, limiting the structure-determination options. Microfocus beamlines have proven to be invaluable in allowing investigators to determine structures from microcrystalline samples (Cherezov *et al.*, 2007 ▸; Coulibaly *et al.*, 2007 ▸; Nelson *et al.*, 2005 ▸; Rasmussen *et al.*, 2007 ▸, 2011 ▸; Warne *et al.*, 2008 ▸). Likewise, next-generation ‘diffraction-limited light sources’ that will produce brighter X-ray beams than current third-generation synchrotron sources (Biasci *et al.*, 2014 ▸; Borland, 2013 ▸; Decker, 2014 ▸; Einfeld, 2014 ▸; Tanaka, 2014 ▸) and XFEL sources hold the promise of an even greater impact on such systems (Barends *et al.*, 2014 ▸; Boutet *et al.*, 2012 ▸; Chapman *et al.*, 2011 ▸; Johansson *et al.*, 2012 ▸, 2013 ▸; Kern *et al.*, 2012 ▸, 2013 ▸, 2014 ▸; Kupitz *et al.*, 2014 ▸; Liu, Wacker *et al.*, 2013 ▸; Redecke *et al.*, 2013 ▸; Sawaya *et al.*, 2014 ▸).

Despite continuing advances in beamline technology, the harvesting and delivery of microcrystals often remains a challenge. Delivery systems have presently focused on two primary approaches, continuous-stream liquid-injection jets and fixed-target systems, each of which offers certain advantages and disadvantages for microcrystal harvesting and analysis. In considering their utility for microcrystal studies, fixed-target devices, such as loops, micromeshes, grids and microfluidic chips, typically offer low sample consumption and relatively high (>30%) automated hit rates (Hunter *et al.*, 2014 ▸). However, loops and micromeshes require sample cryoprotection to prevent rapid dehydration (Cohen *et al.*, 2014 ▸; Hirata *et al.*, 2014 ▸). The cryoprotection process is frequently time-consuming and can influence the structures obtained for crystallized targets (Fraser *et al.*, 2009 ▸, 2011 ▸; Halle, 2004 ▸). Other grid or chip-based fixed-target approaches allow data collection at room temperature, but they also generally require either that crystals be grown and analyzed in a single, highly specialized type of device (Cipriani *et al.*, 2012 ▸; Hansen *et al.*, 2006 ▸; Khvostichenko *et al.*, 2014 ▸) or that crystals be transferred into non-native harvesting conditions such as oil (Hunter *et al.*, 2014 ▸). An ideal sample-delivery platform would allow crystals grown in any format to be transferred and analyzed in a gentle and native-like manner so as to avoid damage caused by a change in solution conditions or mechanical stress. At the same time, such a device would localize microcrystals in defined, addressable locations to optimize the efficiency of sample usage during data collection.

Here, we report the development of a new chip that has minimal X-ray background scattering and absorption properties. The chip exhibits a three-layer design consisting of silicon nitride observation windows, a photoresist microcrystal support and a Kapton film cover (Figs. 1 ▸ *a* and 1 ▸ *b*). Loading of the chips is performed by simply pipetting a sample solution onto the observation windows and laying the Kapton film over the drop. Very little material (<2 µl) is required for loading, and microcrystals are maintained in their native growth conditions as they are transferred to the device. The Kapton film and silicon nitride layers mitigate the effects of dehydration, allowing ample time for data collection from the chips using manual targeting. Long-term approaches to create an airtight seal for microcrystals within the devices to enable the temporary storage and shipping of samples are presently under investigation.

Similar to micromeshes and grids (Cohen *et al.*, 2014 ▸), exposed silicon nitride grids (Hunter *et al.*, 2014 ▸) and Kapton film-based grids (Zarrine-Afsar *et al.*, 2012 ▸), the materials in the device presented here and their thin nature (<15 µm total chamber thickness for photoresist stripe chips) offer high X-ray transmission and allow crystals to be positioned in random orientations. Accordingly, the silicon nitride chips were used to obtain a complete diffraction data set at a low exposure dose, collecting a series of still images from hundreds of single HEWL microcrystals to simulate XFEL-type diffraction data. HEWL microcrystals have been used for the validation of liquid-jet-based (Botha *et al.*, 2015 ▸), fixed-target-based (Coquelle *et al.*, 2015 ▸; Lyubimov *et al.*, 2015 ▸) and capillary-based (Stellato *et al.*, 2014 ▸) delivery systems. From data collected on HEWL microcrystals loaded in the chips, a structure to the full extent of resolution and quality expected for the HEWL model system was determined. The data set consistently contained single images exhibiting high-resolution diffraction peaks, often to 1.3 Å resolution (Supplementary Figs. S3*b* and S6). The native data were of sufficient quality to determine and refine the structure of HEWL to 1.55 Å resolution using molecular replacement. Notably, the structure of HEWL was determined using only 324 collected diffraction images, which is on the order of a microcrystalline sample that could be obtained from a single hanging-drop vapor-diffusion experiment. It is thus possible that silicon nitride-based devices, together with state-of-the-art serial data-reduction software, would make the extraction of high-quality diffraction data from crystals obtained during sparse-matrix crystallization trials feasible.

The results further suggest that the multi-layer chip should be suitable for even smaller (<10 µm) microcrystal targets, as well as for microcrystals of complexes with large unit-cell parameters. The high quality of the data indicates that the diffraction obtained from the chips should be sufficient for tasks that require high signal-to-noise measurements, such as native sulfur SAD phasing (Liu, Liu *et al.*, 2013 ▸). In this regard, the top Kapton film layer of the chips could be replaced by an even lower *Z*-score/higher X-ray transmission material if necessary, such as another silicon nitride window, beryllium or potentially graphene, to promote such studies (Kraus *et al.*, 2014 ▸; Meyer *et al.*, 2007 ▸). Efforts towards the design of such devices are currently under way.

Test XFEL exposures of HEWL microcrystals loaded into the silicon nitride chips suggest the utility of the device for future X-ray diffraction experiments using an XFEL beam source (Supplementary Fig. S3*a*). While the exposure of photoresist-patterned silicon nitride devices to an unattenuated 3 µm beam produced a hole in the Kapton film and a ring of melted photoresist approximately 100 µm in diameter (Supplementary Figs. S4*a* and S4*b*), the diffraction data quality appeared to be unaffected (Supplementary Fig. S3*a*). Additionally, XFEL beam exposures approximately 250 µm from the preceding shot still produced high-quality lysozyme diffraction patterns (not shown), indicating that efficient data collection is possible using rastering approaches. Also, no noticeable dehydration occurred as a result of the damage sustained while the chip was mounted for X-ray diffraction experiments using a 3 µm XFEL beam. By comparison, exposure with a 30 µm beam did not affect the Kapton film or the photoresist structures surrounding the interaction point with the beam in any observable manner (Supplementary Figs. S4*c* and S4*d*).

A remaining challenge in microcrystal delivery systems is to maximize sample hit rates so that close to 100% of the images acquired during the automated collection of diffraction data contain a usable diffraction pattern from a single microcrystal. A device capable of creating a fully addressable array of microcrystals captured from dilute solutions has been developed as a valuable step towards this goal (Lyubimov *et al.*, 2015 ▸). Recent work involving silicon nitride-based devices (Coquelle *et al.*, 2015 ▸; Frank *et al.*, 2014 ▸; Hunter *et al.*, 2014 ▸; Kimura *et al.*, 2014 ▸) has demonstrated the utility of silicon nitride as a low-backscatter material for use with weakly diffracting microcrystalline samples. However, these recent devices have not featured a means of influencing microcrystal location. Here, microcrystal location has been favorably biased through the creation of alternating hydrophobic/hydrophilic surfaces (Fig. 4 ▸). Although crystals were not perfectly positioned at defined locations, HEWL microcrystals could be preferentially localized to photoresist features that had been rendered hydrophilic through oxygen plasma treatment. Efforts to put all of these advances together into a highly efficient crystal-harvesting system are currently under way.

Furthermore, now that a combination of materials (silicon nitride, photoresist and polyimide film) and microfabrication approaches (oxygen plasma treatment) that facilitates diffraction data collection while minimizing backscatter and influences the positioning of microcrystals have been established, this platform can be used to design chips for a variety of micro-manipulation experiments, such as heavy-atom soaking. In the future, it is expected that the collection of data sets requiring large numbers of images, such as *ab initio* phasing from native sulfur SAD experiments, will be greatly aided by an ability to position microcrystals at defined, targetable locations that can be programmed into an automated data-collection system. Further improvement of surface properties and feature dimensions in the three-layer devices described here should help to facilitate such goals.

## Supplementary Material

PDB reference: hen egg-white lysozyme, 4z98

## Figures and Tables

**Table 1:** Data collection, merging statistics and structure refinement Values in parentheses are for the outer shell.

Data-collection and merging statistics	
No. of crystals	324
Wavelength ()	1.03318
Resolution range ()	39.591.55 (1.581.55)
Space group	*P*4_3_2_1_2
Unit-cell parameters (, )	*a* = *b* = 79.14, *c* = 38.11, = = = 90.0
Total reflections	138868
Unique reflections	17566 (817)
No. of observations†	7.90 (3.44)
Completeness (%)	96.9 (91.6)
Mean *I*/(*I*)	14.58 (3.68)
Wilson *B* factor (^2^)	19.96
CC_1/2_ (%)	82.2 (15.8)
Structure-refinement statistics	
*R*_work_	0.205
*R*_free_	0.246
R.m.s.d., bonds ()	0.008
R.m.s.d., angles ()	1.08
Ramachandran favored (%)	96
Ramachandran allowed (%)	3
Ramachandran outliers (%)	0

† Corresponds to the number of partial observations per Miller index as reported by *PRIME* (Uervirojnangkoorn *et al.*, 2015 ▸).
